# A tissue-engineered humanized xenograft model of human breast cancer metastasis to bone

**DOI:** 10.1242/dmm.014076

**Published:** 2014-02

**Authors:** Laure Thibaudeau, Anna V. Taubenberger, Boris M. Holzapfel, Verena M. Quent, Tobias Fuehrmann, Parisa Hesami, Toby D. Brown, Paul D. Dalton, Carl A. Power, Brett G. Hollier, Dietmar W. Hutmacher

**Affiliations:** 1 Regenerative Medicine Group, Institute of Health and Biomedical Innovation, Queensland University of Technology, 60 Musk Avenue, Kelvin Grove, Brisbane, QLD 4049, Australia.; 2 Biotec TU Dresden, Tatzberg 47/49, 01307 Dresden, Germany.; 3 Orthopedic Center for Musculoskeletal Research, University of Wuerzburg, Brettreichstrasse 11, 97072 Wuerzburg, Germany.; 4 Department of Obstetrics and Gynecology, University Hospital Erlangen, Friedrich-Alexander University Erlangen-Nuremberg, Universitaetsstrasse 21–23, 91054 Erlangen, Germany.; 5 Donnelly Centre for Cellular & Biomolecular Research, University of Toronto, 160 College Street, Toronto, Ontario M5S 3E1, Canada.; 6 Biological Resources Imaging Laboratory, Mark Wainwright Analytical Centre, Lowy Cancer Research Centre, University of New South Wales, Randwick, NSW 2052, Australia.; 7 Australian Prostate Cancer Research Centre, Translational Research Institute, 37 Kent Street, Woolloongabba, Brisbane, QLD 4102, Australia.; 8 Tissue Regeneration Program, Institute of Health and Biomedical Innovation, Queensland University of Technology, 60 Musk Avenue, Kelvin Grove, Brisbane, QLD 4049, Australia.; 9 George W. Woodruff School of Mechanical Engineering, Georgia Institute of Technology, 801 Ferst Drive Northwest, Atlanta, GA 30332, USA.; 10 Institute for Advanced Study, Technical University Munich, Lichtenbergstrasse 2a, 85748 Garching, Germany.

**Keywords:** Humanized xenograft model, Bone metastasis, Breast cancer, Osteotropism, Tissue engineering, Melt electrospinning

## Abstract

The skeleton is a preferred homing site for breast cancer metastasis. To date, treatment options for patients with bone metastases are mostly palliative and the disease is still incurable. Indeed, key mechanisms involved in breast cancer osteotropism are still only partially understood due to the lack of suitable animal models to mimic metastasis of human tumor cells to a human bone microenvironment. In the presented study, we investigate the use of a human tissue-engineered bone construct to develop a humanized xenograft model of breast cancer-induced bone metastasis in a murine host. Primary human osteoblastic cell-seeded melt electrospun scaffolds in combination with recombinant human bone morphogenetic protein 7 were implanted subcutaneously in non-obese diabetic/severe combined immunodeficient mice. The tissue-engineered constructs led to the formation of a morphologically intact ‘organ’ bone incorporating a high amount of mineralized tissue, live osteocytes and bone marrow spaces. The newly formed bone was largely humanized, as indicated by the incorporation of human bone cells and human-derived matrix proteins. After intracardiac injection, the dissemination of luciferase-expressing human breast cancer cell lines to the humanized bone ossicles was detected by bioluminescent imaging. Histological analysis revealed the presence of metastases with clear osteolysis in the newly formed bone. Thus, human tissue-engineered bone constructs can be applied efficiently as a target tissue for human breast cancer cells injected into the blood circulation and replicate the osteolytic phenotype associated with breast cancer-induced bone lesions. In conclusion, we have developed an appropriate model for investigation of species-specific mechanisms of human breast cancer-related bone metastasis *in vivo*.

## INTRODUCTION

Bone metastasis is one of the most frequent complications of breast cancer (BC), occurring in 80% of patients with advanced disease (Kozlow and Guise, 2005). The preferential homing of circulating cancer cells to the skeleton has been attributed to mechanical factors, such as blood-flow pathways, as well as favorable molecular interactions between the tumor cells and the target bone site (Chambers et al., 2002). For example it has been shown that metastatic cancer cells have the ability to usurp hematopoietic stem cell (HSC) homing pathways to reach the bone microenvironment (Kaplan et al., 2005; Psaila and Lyden, 2009; Shiozawa et al., 2011). Skeletal metastases from BC are usually associated with an increased osteoclast activity in response to multiple growth factors and cytokines produced by the cancer cells. In turn, growth factors released from the degradation of the bone matrix further enhance tumor growth (Ooi et al., 2011). Once overt metastases develop, there are only palliative treatment options available to prevent further disease progression and reduce the associated pain and symptoms (Suva et al., 2011).

A major obstacle to the development of new therapeutic concepts to prevent or treat bone metastases has been the lack of appropriate *in vivo* models that replicate the complexity of the human disease. Different xenograft models have been used to generate human cancer metastasis to bone in small animal models, depending on the stage of the disease to be investigated. Direct injection of human cancer cells into the mouse tibia or femur allows consistent development of bone metastases and can replicate tumor-induced changes in murine bone (Le Gall et al., 2007; Zheng et al., 2008; Ooi et al., 2010). This approach, however, mimics only the final stages of bone colonization by extravasated cancer cells and replicates a primary tumor model rather than a metastasis model. A common method to generate experimental metastasis is the intracardiac injection of osteotropic cancer cells, which quickly induces bone metastases at a high frequency (Yoneda et al., 2001; Henriksen et al., 2002; Yi et al., 2002; Harms et al., 2004; Khalili et al., 2005; Canon et al., 2008). Although these traditionally used xenograft models allow the proliferation of human tumor cells in the mouse skeleton, they are associated with certain limitations. To avoid graft rejection, immune-compromised hosts are necessary, which eliminates the ability to examine the role of the immune system in tumor progression. Moreover, interspecies differences such as incompatibilities in receptor-ligand interactions between the human tumor cells and murine host microenvironment can impair the species-specific pathways occurring during human cancer progression and metastasis (Khanna and Hunter, 2005; Rangarajan and Weinberg, 2003).

**TRANSLATIONAL IMPACT**

**Clinical issue**

Bone metastasis is a life-threatening complication that occurs in 80% of women with advanced breast cancer. The clinical management of patients affected by bone metastases is particularly challenging because early-stage detection is difficult and once overt lesions develop the disease is incurable with currently available treatment options. The development of approaches to prevent or treat bone metastases is hampered by the lack of appropriate animal models to mimic human bone metastatic disease. Traditionally, injection of human cancer cells into mice has been used to investigate bone metastasis but in these models human cancer cells have to disseminate to and grow in murine bone, which does not replicate the physiological tumor-bone interactions that occur in patients. More recently, animal models of human bone metastasis have been developed using subcutaneous implantation of human bone but these models suffer from problems such as donor-related variability and poor viability of the implant.

**Results**

Tissue-engineered systems have the potential to overcome some of the drawbacks of native bone implants and to provide more reproducible and controllable models. In this study, the authors demonstrate that engineered constructs based on biocompatible polymer scaffolds seeded with human bone-forming cells combined with the osteoinductive growth factor bone morphogenetic protein 7 can create a viable ectopic ‘organ’ bone in a mouse model. The newly formed bone microenvironment incorporates human bone cells and human-derived matrix proteins and is therefore humanized. The authors show that human breast cancer cell lines with different affinity for bone metastasize to the human tissue-engineered bone construct (hTEBC) after intracardiac injection. Moreover, the metastases detected in the hTEBC replicate the osteolytic (bone-damaging) phenotype of the bone lesions typically induced by metastasis of breast cancer cells.

**Implications and future directions**

This study establishes an *in vivo* model that mimics the metastasis of human breast cancer cells to a human bone-like microenvironment. The new model provides a platform for the dissection of the molecular mechanisms that control the homing to and growth of human breast cancer cells in human bone and for the development of new treatment strategies for bone metastasis. Future improvements of this model such as the incorporation of additional human-derived components (for example, hematopoietic stem cells) into the engineered bone will allow the replication of not only human bone-tumor interactions but also the species-specific interactions that occur between tumor cells and bone marrow stem cells in the metastatic niche.

Humanized mice are promising translational models for studying human diseases. To create humanized xenograft models of bone metastasis, human fetal or adult bone pieces have been implanted in the flank of immunodeficient mice to serve as a target site for human cancer cells (Shtivelman and Namikawa, 1995; Nemeth et al., 1999; Yonou et al., 2001; Kuperwasser et al., 2005; Yang et al., 2007). This approach represents a great step forward in modeling human bone metastatic disease by allowing the investigation of species- and tissue-specific interactions in a small animal model. However, the use of human bone tissue is associated with practical issues such as availability of tissue, donor-related variability and difficulty in maintaining the functionality and viability of the implant (Holzapfel et al., 2013; Hutmacher et al., 2009). A few recent studies have applied tissue engineering principles to overcome the drawbacks associated with the implantation of native human bone and provide more reproducible, controllable and functional implants (Schuster et al., 2006; Moreau et al., 2007). However, in these models the engineered tissues were not fully characterized from a bone biology point of view. In fact, the histology showed a bone microenvironment with no evident bone marrow and with fibrous tissue filling the spaces between the remaining scaffold structures and the newly deposited mineralized tissue. Moreover, the authors did not demonstrate the human origin of the bone cells and matrix, nor did they show that their models could replicate the osteolytic pattern of bone metastasis.

In the presented study, we used primary human osteoblastic cell (hOB)-seeded melt electrospun scaffolds in combination with recombinant human bone morphogenetic protein 7 (rhBMP-7) to promote the development of a viable humanized bone microenvironment in non-obese diabetic/severe combined immunodeficient (NOD/SCID) mice. The human tissue-engineered bone construct (hTEBC) led to the formation of a mature ‘organ’ bone incorporating human elements. Importantly, the hTEBCs could be applied as a target tissue for the homing of human BC cells injected into the circulatory system. Using this approach, the osteolytic phenotype characteristic of bone metastases from BC could be replicated. We propose that this tissue-engineered platform is a promising tool for elucidating the mechanisms of human BC metastasis to human bone in a murine host.

## RESULTS

### Replicating a humanized bone microenvironment *in vivo* using the hTEBC model

#### *In vitro* characterization of the hTEBCs

**Fig. 1.**
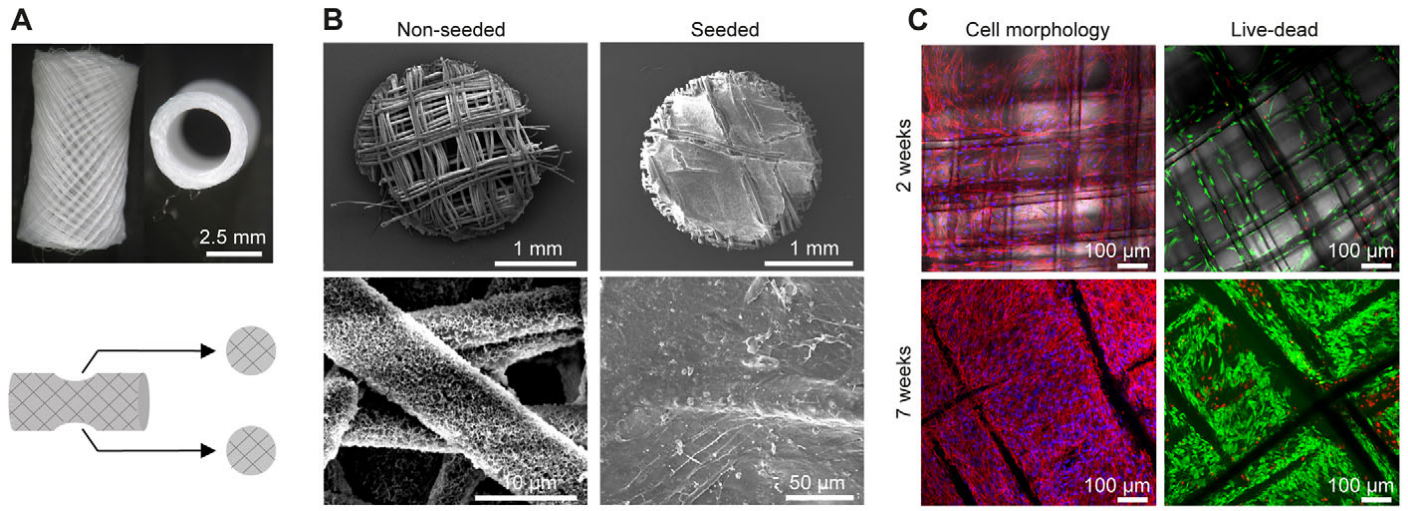
***In vitro* characterization of hTEBCs.** (A) Representative images of the melt electrospun PCL scaffolds and schematic showing how biopsies are retrieved for analysis. (B) Characterization of the CaP-coated scaffolds by SEM before cell seeding and after 7 weeks of culture *in vitro*. (C) Morphology and viability of the hOBs cultured on the scaffolds is assessed by CLSM on cells stained respectively for F-actin (red) and nuclei (blue), or with markers for live (fluorescein diacetate, green) and dead (propidium iodide, red) cells after 2 and 7 weeks of culture *in vitro*.

The underlying melt electrospun polycaprolactone (PCL) scaffolds for the hTEBCs were tubular in shape (10 mm length, 4.7 mm outer and 4 mm inner diameter) and comprised of 30 μm diameter filaments spaced ~0.5 mm apart (Fig. 1A). Biopsies were taken at different stages of the hTEBC preparation (Fig. 1A) to characterize the scaffold surface properties before and after cell seeding, as well as the morphology and viability of the hOB cells prior to *in vivo* application. Scanning electron microscopy (SEM) analysis confirmed the presence of a homogeneous layer of mineralized particles on the scaffold fibers after coating with calcium phosphate (CaP) (Fig. 1B). The pores of the scaffolds were covered by a dense cell layer as shown by SEM (Fig. 1B) after 7 weeks and by confocal laser scanning microscopy (CLSM) at 2 and 7 weeks post-seeding of the hOBs (Fig. 1C). Live-dead staining indicated that more than 90% of the seeded hOB cells were viable (green) at the time of implantation (Fig. 1C).

#### hTEBCs promote mineralized matrix formation

**Fig. 2.**
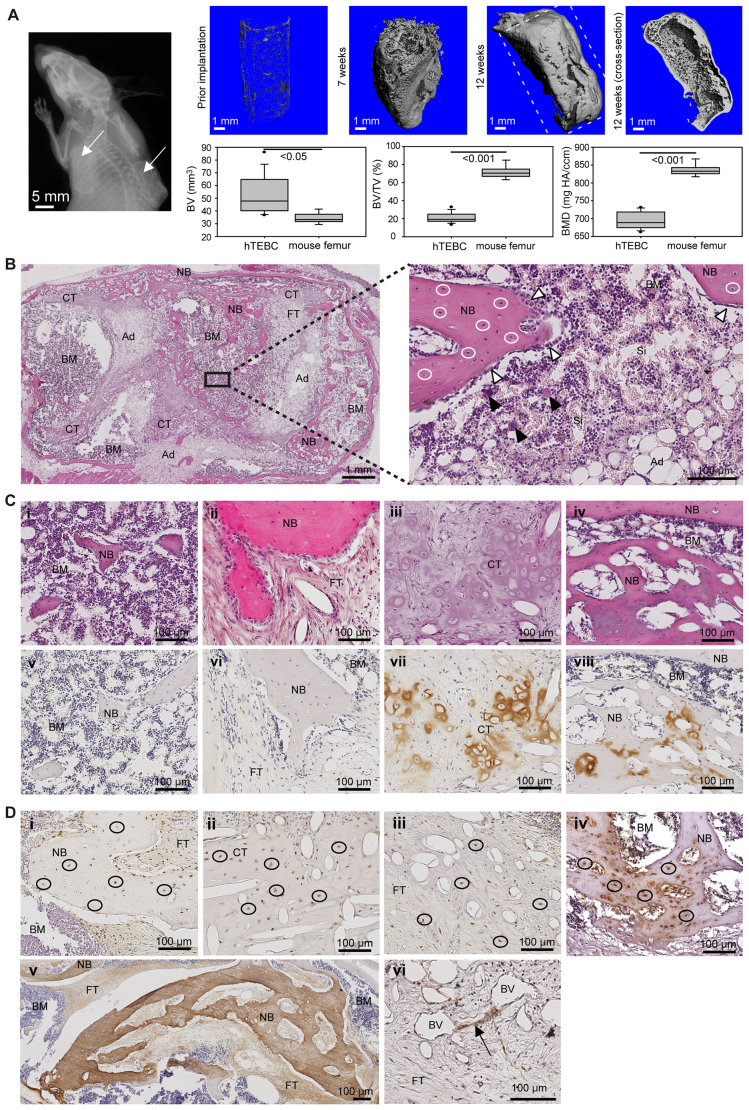
**Application of the hTEBC to replicate a humanized bone microenvironment.** (A) Representative X-ray image at 8 weeks after implantation of the hTEBCs indicates successful bone formation (arrows) in the constructs. Mineralized tissue formation is shown in representative μ-CT reconstructions of hTEBCs prior to implantation (*n*=4) and 7 (*n*=4) and 12 weeks (*n*=10) after implantation. μ-CT quantification of BV, BV/TV and BMD for the hTEBCs (*n*=10) and mice femurs (*n*=5). (B) Representative H&E images showing the bone ossicle at high and low magnification. White circles, live osteocytes; white arrowheads, bone-lining osteoblasts; black arrowheads, megakaryocytes. (C) H&E images indicate that bone formation occurs both via direct intramembranous ossification (i,ii) and endochondral ossification of a cartilaginous matrix (iii,iv). Correlating with the morphological observations, IHC for collagen II (brown stain) shows areas of new bone negative of collagen II (v,vi) and the presence of positive staining in cartilage-like structures and bone (vii,viii). (D) IHC analysis for human NuMA (brown stain) confirms the human origin of cells in the bone (i), cartilage (ii) and fibrous tissue (iii). Osteocytes are also positive for human osteocalcin (brown stain) (iv). Human-derived matrix protein collagen type I (v) is detected mostly in the inner part of the hTEBCs (brown stain). Single human CD146-positive cells (brown stain) are located in the vicinity of blood vessels (vi). Black circles, human cells; black arrow, human CD146-positive cells; NB, new bone; BM, bone marrow; CT, cartilaginous tissue; FT, fibrous tissue; Ad, adipocytes; Si, sinusoids; BV, blood vessel.

After subcutaneous implantation of the hTEBCs in the flanks of NOD/SCID mice, the deposition of mineralized tissue was detected with X-ray imaging in all hTEBCs (Fig. 2A). The implanted scaffolds retained their tubular shape *in vivo* and bone growth was restricted to the scaffold boundaries (Fig. 2A; supplementary material Fig. S1A). Serial three-dimensional (3D) reconstructions of micro-computed tomography (μ-CT) data show that mineralization occurred *in vitro* prior to implantation and progressed over 12 weeks *in vivo*, with mineralized tissue forming both around and inside the cylindrical construct (Fig. 2A). μ-CT quantification of the mineralized tissue volume (BV), mineralized tissue volumetric density (BV/TV) and bone mineral density (BMD) in the hTEBCs and in endogenous mouse femurs is indicated in Fig. 2A. These results demonstrate the formation of a significantly higher volume of mineralized bone matrix in the hTEBCs compared with mouse femurs (*P*<0.05). However, BV/TV and BMD were significantly lower (*P*<0.001) in hTEBCs compared with mouse bones, which correlates with the large cavities filled by soft tissue in the engineered bone (Fig. 2B).

#### Engineering the ‘organ’ bone

Histological analysis of hematoxylin and eosin (H&E)-stained sections from the center of the implant revealed the formation of a mature bone ossicle consisting mainly of an outer shell of cortical-like bone surrounding trabecular structures and bone marrow spaces (Fig. 2B). Other types of tissue that formed in the hTEBCs included cartilage-like tissue, adipose tissue and fibrous connective tissue. Residual PCL scaffold fibers could only be observed in the form of voids left in the tissue. Because the mass loss of PCL scaffolds is negligible in the first 6 months following *in vivo* implantation (Lam et al., 2009a), the absence of the PCL fibers is due to the processing in xylene solution prior to paraffin embedding and not to degradation of the polymer. The newly formed bone was well vascularized (supplementary material Fig. S1B–D) and viable, as shown by the presence of live osteocytes in the bone lacunae (Fig. 2B). The presence of osteocytes, bone-lining osteoblast-like cells (Fig. 2B) and tartrate-resistant acid phosphatase (TRAP)-positive osteoclasts (not shown) indicated that the hTEBC model replicates a normal mature bone microenvironment. Bone marrow spaces contained hematopoietic cells, adipocytes and numerous sinusoids (Fig. 2B). The cellular composition of the bone marrow microenvironment included hematopoietic cells of different lineages and differentiation stages such as erythroid and megakaryocytic cells (Fig. 2B; supplementary material Fig. S2), as can be found in the bone marrow in the long bones of mice (Travlos, 2006).

#### Replication of intramembranous and endochondral ossification pathways

Histological analysis of the hTEBCs showed evidence of bone formation via both the intramembranous and endochondral ossification pathways. The H&E staining revealed the presence of isolated bone spicules surrounded by a cluster of cells or immature fibrous tissue (Fig. 2Ci,ii). These stained negatively for collagen type II using immunohistochemistry (IHC) (Fig. 2Cv,vi), thus indicating that the bone formation process occurred without a cartilage intermediary and via intramembranous ossification. However, collagen II-positive cartilage-like structures could be visualized, mainly in the center of the hTEBCs (Fig. 2Ciii,vii). Collagen II-positive tissue was also found surrounded by calcified bone matrix, thus indicating that bone formation in the hTEBCs replicated the different stages of endochondral ossification (Fig. 2Civ,viii). Analysis of H&E staining and cartilage-specific collagen II deposition has been used previously to draw conclusions on the ossification type of tissue-engineered bone (Tortelli et al., 2010).

#### Humanized model

IHC analysis of the hTEBCs with human-specific antibodies was used to determine the species of origin of the cells in the newly formed tissues. A large proportion of the cells in the fibrous and cartilage-like tissue were positive for human nuclear mitotic apparatus protein 1 (NuMA) (Fig. 2Dii,iii). In the bone matrix only some of the osteocytes were NuMA-positive (Fig. 2Di), which might be explained by the fact that this marker is usually not expressed in non-proliferating or end-differentiated cells (Sun and Schatten, 2006). However, embedded osteocytes were shown to express the late-stage bone marker osteocalcin stained with a human-specific antibody (Fig. 2Div). Moreover, IHC against human collagen type I and human osteocalcin also demonstrated the presence of human-derived bone matrix proteins in the newly formed bone (Fig. 2Div,v). Positive staining for human collagen I and osteocalcin was located mostly in the center of the scaffolds, whereas the thin cortex-like shell of lamellar bone surrounding the scaffold was negative. Using an antibody specific for human CD146, the presence of single human-derived mesenchymal progenitor cells, as defined by Sacchetti et al. (Sacchetti et al., 2007), was detected in perivascular regions (Fig. 2Dvi) as previously described (Tormin et al., 2011). As expected, staining for hematopoietic cells, endothelial cells and osteoclasts was negative using the human-specific antibodies, indicating that these cells were of murine origin. From these stainings, we conclude that the hTEBCs contained large areas of human bone components, such as human bone cells and human-derived bone matrix, whereas blood vessels and the bone marrow were derived from the host. Sections of mouse femur were used as negative controls to rule out any cross-reactivity of the antibodies with mouse tissue, and human bone sections served as positive controls (supplementary material Fig. S3).

### hTEBCs are a target site for metastasis by bone-seeking BC cells

**Fig. 3.**
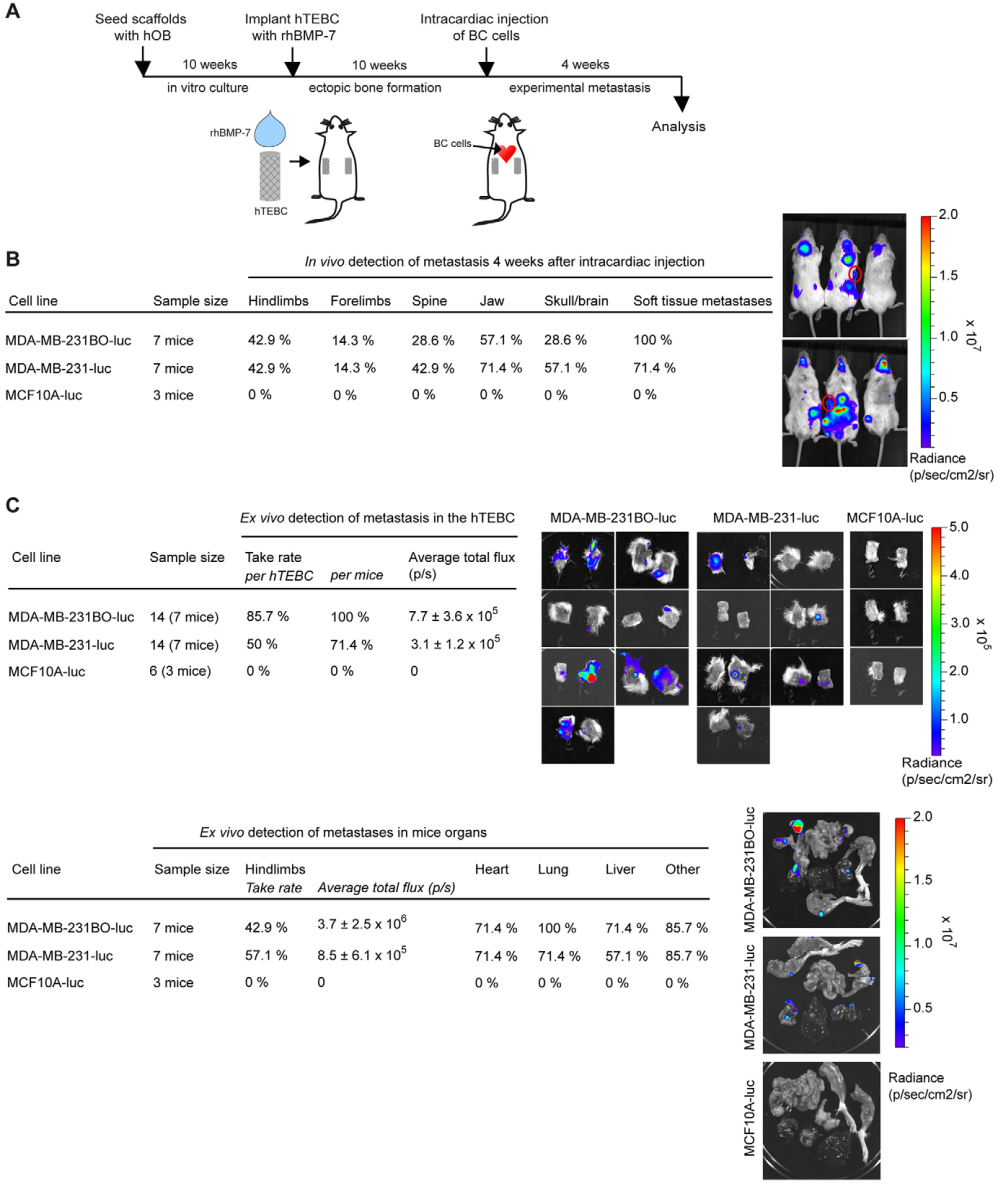
**Application of the hTEBC as a target site for BC metastasis.** (A) Schematic of the experimental design. (B) Results and representative bioluminescence-photographic overlay images showing metastases to organs and hTEBCs (red circles) detected by *in vivo* BLI at 4 weeks after intracardiac injection. (C) Tables summarizing metastases detected by *ex vivo* BLI in the hTEBCs and mice organs. Images of the hTEBCs and representative images from mice organs are shown.

After having established that a humanized bone microenvironment could be generated in NOD/SCID mice, we set out to test the potential of hTEBCs as target sites for human BC cells. Therefore, the human BC cell line MDA-MB-231, a bone-seeking substrain (MDA-MB-231BO) and a non-metastatic control cell line (MCF10A) were transfected to express luciferase and injected into the left ventricle of the mice heart (Fig. 3A). *In vivo* bioluminescence imaging (BLI) indicated that the first metastases developed in the host 1 week after intracardiac injection of the BC cells (not shown). However, a precise estimation of the onset of metastases within the hTEBCs was hampered by the fact that the relatively high bioluminescent signals originating from the organs during whole-body live imaging screened the weaker signals from the hTEBCs. Extensive multiorgan metastasis could be detected after 4 weeks in the metastatic cell groups but not in the MCF10A control group (Fig. 3B). Skeletal metastases were seen predominantly in the regions of the jaw, hind limbs and spine. The mice started to manifest signs of hind limb paralysis, difficulty in breathing and cachexia at 4 weeks after intracardiac injection of the BC cells, and the experiment was ended. After sacrifice of the mice, hTEBCs and organs were dissected and imaged *ex vivo* (Fig. 3C). A high homing rate of the metastatic cell lines to the hTEBCs was observed, with the detection of bioluminescent signal in 85.7% of hTEBCs in 100% of mice for the MDA-MB-231BO cells and 50% of hTEBCs in 71.4% of mice for the MDA-MB-231 group. The average total flux of bioluminescent signal from the bone-seeking MDA-MB-231BO cells was higher than for the parental MDA-MB-231 cells in both hTEBCs and mice hind limbs, but the differences were not statistically significant (*P*>0.05). BC cells metastasized less frequently to mice hind limbs compared with hTEBCs, but the average total flux measured in mice hind limbs was higher, although not significantly (*P*>0.05). *Ex vivo* analysis also indicated the presence of metastases in the mice hearts, lungs, livers and several other soft tissue organs for the metastatic cell groups. No signal was seen in the hTEBCs or mice organs for the MCF10A control group.

### The hTEBC model reproduces features characteristic of human BC-related bone metastasis

**Fig. 4.**
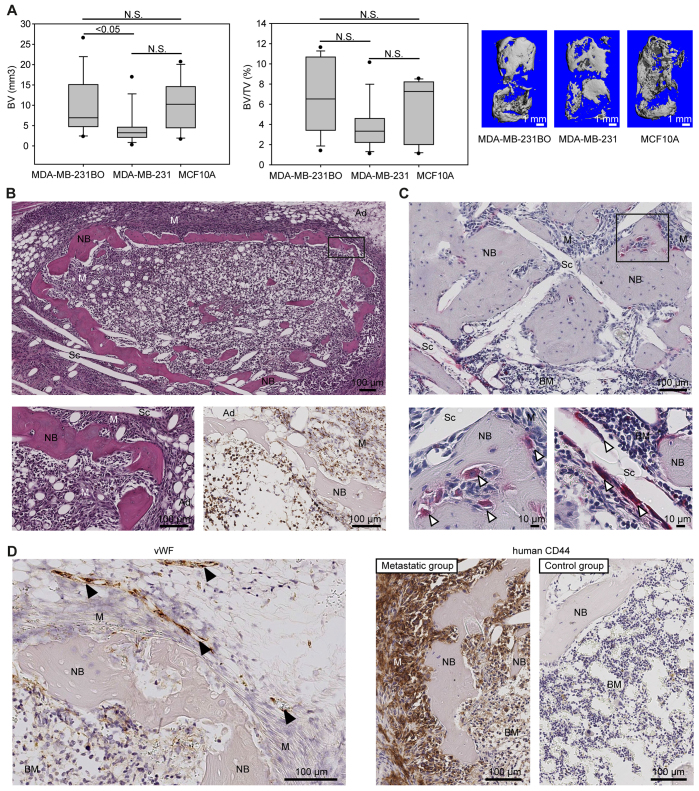
**Application of the hTEBC replicates features of BC-related bone metastasis.** (A) μ-CT quantification of BV and BV/TV with representative 3D reconstructions of the hTEBCs for the mice injected intracardially with MDA-MB-231BO (*n*=14), MDA-MB-231 (*n*=14) and MCF10A (*n*=6) cells. (B) Representative H&E images show a metastasis in the newly formed bone at high and low magnification. IHC stain for human mitochondria (brown stain) confirms the human origin of the malignant cells in the hTEBCs. (C) TRAP staining (red stain) reveals that osteoclasts actively degrade the bone in contact with the BC cells and are also found adjacent to the voids left by the scaffold fibers. (D) IHC analysis shows the presence of vWF-positive blood vessels adjacent to the bone metastases. Metastatic cancer cells express human CD44; no human CD44-positive cells are detected in controls. White arrowheads, osteoclasts; black arrowheads, blood vessels; M, metastases; NB, new bone; BM, bone marrow; Ad, adipocytes; Sc, scaffold.

Analysis by μCT indicated a lower median BV and BV/TV in the hTEBCs for the metastatic cell groups compared with the control group; however, the differences were not significant (Fig. 4A). Thus, paraffin-embedded samples were serially sectioned and stained with H&E. Histological analysis revealed the presence of micrometastases in the hTEBCs from the metastatic cell groups, where the cancer cells surrounded the newly formed bone and invaded the marrow spaces (Fig. 4B). IHC using an antibody specific for human mitochondria confirmed the human origin of the malignant cells in contact with the newly formed humanized bone (Fig. 4B). The bone surface in contact with the cancer cells displayed an irregular profile, with resorption pits indicating degradation of the matrix by osteoclasts. TRAP staining confirmed the presence of osteoclasts actively degrading the bone (Fig. 4C), thus reflecting the osteolytic character of metastatic BC. TRAP-positive osteoclasts were also detected along the voids left by the scaffold fibers in the presence of the tumor cells (Fig. 4C). Isolated cancer cells were also seen adjacent to voids left by the scaffold fibers (not shown). IHC against von Willebrand factor (vWF) detected the presence of blood vessels in the metastases along the bone (Fig. 4D). The metastatic cancer cells were shown by IHC to express human CD44 (Fig. 4D).

## DISCUSSION

Previous studies have utilized tissue-engineered constructs as an alternative to human bone chips for the development of humanized bone metastasis models in mice (Schuster et al., 2006; Moreau et al., 2007). Although these approaches have been successful in demonstrating that engineered microenvironments can be used as target sites for metastatic spread and growth, they lacked evidence of the formation of a mature ‘organ’ bone, the presence of a humanized microenvironment or the replication of cancer-induced bone degradation.

We hypothesized that implanting hOB-seeded polymer scaffolds in mice in combination with rhBMP-7 would allow the replication of a mature and viable human bone microenvironment. Compared to bone chips, the hTEBCs have a distinct advantage in allowing control of the shape, size and surface properties of the implant as well as the opportunity to characterize the cell viability prior to implantation. The hTEBCs integrated well under the skin and did not result in wound problems or dehiscence that are common after implantation of sharp-edged bone pieces (Scepansky et al., 2001). The hTEBCs led to the formation of mineralized tissue in all implants, indicating a high reproducibility for this procedure. Mineralization was initiated *in vitro* when hOB cells were cultured under osteogenic conditions and progressed over time after *in vivo* implantation, yielding an amount of mineralized tissue that was comparable or higher than obtained in other recent models of ectopic bone development (Kempen et al., 2010; Song et al., 2010). Compared to mouse femurs, the hTEBCs led to more mineralized tissue formation, but the overall density of the tissue was lower. In a previous study on 242 human cancellous bone specimens from 70 donors and four different skeletal sites, the values measured for BV/TV ranged from ~5 to 30% (Ulrich et al., 1999). Thus, the mineralized tissue fraction observed in the hTEBCs more closely mimics the structure of human bone than endogenous mouse bones. Bone is a complex and dynamic organ that incorporates different hematopoietic and non-hematopoietic cell types, proteins and growth factors in addition to the mineralized matrix, and undergoes constant remodeling. The hTEBC was able to reproduce not only the mineralized tissue component, but a viable and functional ‘organ’ bone comprising live bone cells, bone marrow and adequate vascularization.

There is increasing evidence that the bone microenvironment is able to actively promote BC cell colonization through the same pathways that are used for the homing of normal HSCs to the bone marrow (Psaila and Lyden, 2009). Thus, replicating a functional HSC niche is important in developing a physiological model of bone metastasis. Using progenitor cells transplanted under the adult mouse kidney capsule, Chan et al. demonstrated that endochondral ossification through the calcification of a cartilage intermediate is essential for the formation of an HSC niche in ectopic bone ossicles (Chan et al., 2009). Several studies have used BMPs for the induction of endochondral bone formation after subcutaneous implantation in mice and shown the recruitment of HSCs to these ectopic bone ossicles (Sasano et al., 1993; Kawai et al., 1994; Krebsbach et al., 2000; Rutherford et al., 2002; Tsumaki and Yoshikawa, 2005; Retting et al., 2009). However, in other engineered ectopic *in vivo* bone models using stem cells and CaP-based scaffolds bone formation occurred via an intramembranous mineralization process and also led to the formation of bone marrow compartments (Eyckmans et al., 2010; Chai et al., 2012). In line with these previous reports, we show evidence that hTEBCs with hOBs and rhBMP-7 replicate both pathways of bone formation that are physiologically found in the skeleton. IHC analysis demonstrated the presence of matrix-embedded osteocytes of human origin as well as human-derived collagen I and osteocalcin in the hTEBCs, thus indicating that the hOBs survived the implantation and were able to form mature bone and differentiate into osteocytes. Moreover, the presence of human spindle-shaped cells in the fibrous tissue and human chondrocyte-like cells in the cartilaginous tissue implies that the donor and not the host cells mainly participated in the endochondral bone formation. This finding is consistent with previous observations where osteoblastic cells obtained from explant cultures of adult human trabecular bone fragments displayed the ability to differentiate into multiple mesenchymal lineages under controlled culture conditions (Nöth et al., 2002). In particular, the cells were able to undergo chondrogenesis *in vitro* (Nöth et al., 2002) and *in vivo* (Gundle et al., 1995).

Our group has extensively characterized the ability of hOBs to differentiate into mature osteoblasts and produce a mineralized extracellular matrix containing proteins and growth factors characteristic of the bone matrix when cultured under osteogenic conditions (Reichert et al., 2010; Taubenberger et al., 2013). However, the presence of human fibroblast and chondrocyte-like cells in the hTEBCs suggests that a fraction of mesenchymal progenitor-like cells persists in the implanted cell population, accounting for the development of human-derived mesenchymal tissues other than bone. This is supported by the detection of single human CD146-positive mesenchymal progenitor cells in the hTEBCs. Moreover, Asahina et al. observed that rhBMP-7 treatment on primary cells obtained through explant culture of newborn rat calvaria induced chondrogenesis *in vitro* by acting on a primitive mesenchymal osteoprogenitor cell subpopulation, but did not appear to reverse committed osteoblasts (Asahina et al., 1993). In a later study, Tortelli et al. also demonstrated that the ossification type of the engineered bone in their model depended strictly on the nature and commitment of the seeded cells (Tortelli et al., 2010).

IHC-positive staining of the human-derived bone matrix proteins collagen type I and osteocalcin was mostly located in the center of the bone organ, indicating that the outer cortical-like shell surrounding the scaffold is a chimeric tissue comprising both donor and host-derived extracellular matrix. Similar findings have been described in a previous study where BMP-7-expressing human gingival fibroblasts were used to engineer bone marrow-containing ossicles (Krebsbach et al., 2000). IHC against human and murine osteonectin, as well as genomic *in situ* hybridization with human-specific Alu probes, demonstrated that the bone was predominantly of human origin in the central region of the transplant and to a lesser extent in the peripheral cortical bone (Krebsbach et al., 2000). Finally, in addition to the replication of a physiological and functional ‘organ’ bone, the hTEBCs provide a humanized bone microenvironment for the investigation of BC cell dissemination.

We hypothesized that the engineered bone obtained with our model could be applied as a target site for metastasis by malignant BC cells injected intracardially. *In vivo* imaging allowed monitoring of the development and growth of multiorgan metastases over 4 weeks in mice. The mice were sacrificed when they started to display signs of illness characteristic of metastatic BC disease, such as hind limb paralysis from spinal metastases, labored breathing due to lung tumors or significant weight loss. When the hTEBCs were imaged individually *ex vivo*, successful dissemination of the metastatic BC cell lines to the scaffolds was observed. To our knowledge this is the first xenograft model of metastasis using the intracardiac injection of BC cells in combination with engineered bone, although this inoculation technique is widely used in traditional xenograft models of metastasis to the mouse skeleton. To date, only one study has combined an ectopic human bone chip model with the intracardiac inoculation of BC cells. Ling et al. showed that injection of 1×10^6^ MDA-MB-231 cells in the left cardiac ventricle of SCID/berg female mice resulted in metastasis to implanted human bone chips in 20% of mice (Ling et al., 2008). In traditional xenograft models, MDA-MB-231 cells metastasize to the skeleton in about 30% of mice and the cells also spread to other organs, thus creating additional morbidity (Sasaki et al., 1995; Li et al., 2001; Yoneda et al., 2001; Kang et al., 2003). In our model we observed that the MDA-MB-231 cells homed to 50% of hTEBCs in 71.4% of mice, thus obtaining a higher take rate than previous studies. Numerous metastases to the mouse skeleton and other soft tissue organs were also detected. The bone-seeking MDA-MB-231BO cells were originally isolated by Yoneda et al. through repeated *in vivo* passaging of the parental MDA-MB-231 cells until they metastasized exclusively to the skeleton in 100% of mice, as determined histologically (Yoneda et al., 2001). In line with these results, MDA-MB-231BO cells exhibited a higher take rate than the parental cells in our model, with metastases detected in 85.7% of hTEBCs in 100% of mice and presenting a higher average bioluminescent signal.

However, metastasis of MDA-MB-231BO cells was not specific to the humanized or mouse bone as the cells also spread to numerous other mouse organs. This observation can be explained by different factors. First, it is known that repeated *in vitro* passaging or variation in culture methods can lead to alterations in the biological behavior of cells, and unexpected changes in the metastatic patterns of cancer cell lines have been described previously (El-Mabhouh et al., 2008). Second, the immune deficiency of each mouse strain (i.e. nude versus NOD/SCID mice) can affect the frequency of metastasis and must be taken into account when comparing experimental observations in xenografts (Pearson and Pouliot, 2012). Finally, the higher incidence rate described in our model might derive from the more sensitive detection method of BLI versus histological analysis. Although both metastatic BC cell lines disseminated more frequently to hTEBCs compared to mice hind limbs, the metastatic process was not specific to the humanized bone microenvironment in our model. Previous studies with humanized xenograft models of BC bone metastasis using human bone chips reported a species-specificity of cancer osteotropism (Kuperwasser et al., 2005; Yang et al., 2007; Lam et al., 2009b). However, this conclusion was often based on the screening of the mouse skeleton for metastases using the less-sensitive histological or X-ray-based detection methods. In a more recent study by Liu et al. using the same cell line, metastasis to both human and mouse bone was detected by whole-body BLI, which contradicted the group’s earlier result (Kuperwasser et al., 2005; Liu et al., 2009).

In this work, we confirmed that hTEBCs support metastasis by human BC cells injected into the blood circulation via intracardiac inoculation. We hypothesized that the metastases in our engineered bone would replicate features of BC-induced bone lesions. MDA-MB-231 and MDA-MB-231BO cells typically induce osteolytic metastases (Yoneda et al., 2001). In hTEBCs, the presence of human metastatic BC cells could be detected in the newly formed bone. Moreover, the irregular profile of the bone surface and the presence of TRAP-positive multinucleated cells in resorption pits indicated that osteoclasts were actively resorbing the bone in contact with the tumor cells. The osteoclasts also displayed a predilection for the surface of the CaP-coated scaffold fibers, and isolated cancer cells were seen invading the engineered tissues along the scaffold fibers. In fact, the bone inorganic component CaP has been shown to promote the adhesion and growth of metastatic tumor cells (Pathi et al., 2011). Blood vessels were detected adjacent to the bone invaded by metastatic cells, indicating that the BC cells could have reached the bone by arresting and extravasating from those vessels and that the blood supply promoted their development. Finally, the metastatic BC cells in the hTEBCs expressed human CD44, an adhesion receptor for the extracellular matrix proteins hyaluronan and osteopontin, which is known to promote cancer cell migration, invasion and metastasis (Hiraga et al., 2013).

The hTEBC is a controllable and reproducible system that supports the development of a physiological and functional humanized ‘organ’ bone and that can be implemented as a target site for the homing of human BC cells and development of osteolytic metastases. Compared with traditional xenograft models of metastasis, hTEBCs recapitulate more closely the structural characteristics of human trabecular bone than mouse femurs and are more frequently colonized by circulating BC cells. Thus, the hTEBC provides a suitable humanized xenograft model for replication of species-specific human BC-related bone metastasis *in vivo* and could be applied in preclinical research to investigate the mechanisms underlying BC homing and colonization as well as for developing new treatment strategies. Although the current approach only incorporates human-derived bone cells and extracellular matrix, the use of an engineered construct provides the opportunity to manipulate the hTEBC in the future, for example by including human endothelium or hematopoietic cells, to eventually further humanize the model.

## MATERIALS AND METHODS

### Cell culture

hOBs were isolated from bone samples obtained under informed consent from female patients undergoing hip or knee replacement surgery (Queensland University of Technology Research Ethics approval number 0600000232) and cultured as described previously (Reichert et al., 2010). MDA-MB-231 cells were purchased from the American Type Culture Collection (ATCC, Manassas, VA). MDA-MB-231BO cells were kindly provided by the University of Texas Health Science Center at San Antonio (San Antonio, TX) (Yoneda et al., 2001). The cells were maintained in high glucose Dulbecco’s modified Eagle medium (DMEM, Life Technologies, Mulgrave, Victoria, Australia) supplemented with 10% fetal bovine serum (FBS, Life Technologies), 100 IU/ml penicillin (Life Technologies), 100 μg/ml streptomycin (Life Technologies) and 1× Glutamax (Life Technologies). MCF10A cells were from the ATCC and cultured in DMEM/F12 (Life Technologies) supplemented with 10% FBS, 20 ng/ml epidermal growth factor (Sigma-Aldrich, St Louis, MO), 100 ng/ml cholera toxin (Sigma-Aldrich), 0.01 mg/ml bovine insulin (Sigma-Aldrich), 500 ng/ml hydrocortisone (Sigma-Aldrich) and penicillin/streptomycin. The cell lines were transduced to express luciferase using a pLenti6/V5-D-TOPO (Life Technologies) lentivirus and selected for at least 2 weeks using Blasticidin (InViVoGen, San Diego, CA) under the ethics approval number 1100000252 for work with genetically modified organisms.

### hTEBC preparation

Medical grade PCL CAPA 6500C (Perstorp, Warrington, Cheshire, UK) with a molecular weight of 50 kDa was used to produce tubular scaffolds by melt electrospinning writing on a custom-built machine as previously described (Brown et al., 2012). The scaffolds were coated with CaP to promote cell attachment, following an established protocol (Vaquette et al., 2013). The scaffolds were sterilized, seeded with 200,000 hOBs and cultured as described previously until they were overgrown with hOBs (Brown et al., 2012). To induce differentiation, the tubes were then transferred from static cultures to the vessel of a bi-axial rotating bioreactor (Singh et al., 2005) containing 500 ml osteogenic media consisting of culture media supplemented with 50 μg/ml L-ascorbic acid-2-phosphate (Sigma-Aldrich), 10 mM β-glycerophosphate (Sigma-Aldrich) and 0.1 μM dexamethasone (Sigma-Aldrich). The bioreactor was placed in a heat-sterilized incubator (Binder, Tuttlingen, Germany) to culture the cells at 37°C and 5% CO_2_ while the vessel rotated continuously along two independent axes to improve fluid flow. The medium was changed every 2 weeks. After 2 months, cultured scaffolds were removed from the bioreactor and 2 mm biopsy punches (Kai Medical, Tokyo, Japan) were used to stamp out small disks from the scaffolds for subsequent cell characterization. For *in vivo* investigations, each scaffold received 30 μl of rhBMP-7 (1 μg/μl) (Olympus Biotech Corporation, Hopkinton, MA) in combination with 60 μl of human thrombin (TISSEEL Fibrin Sealant, Baxter Healthcare International, Deerfield, IL) into the tubular cavity, before the addition of 60 μl of human fibrinogen to induce fibrin gel formation (TISSEEL Fibrin Sealant, Baxter Healthcare International). Constructs were kept on ice until implantation into mice.

### *In vitro* characterization

Surface analysis of the hTEBC was performed by SEM, and cell morphology was assessed by CLSM using a SP5 confocal microscope (Leica, Wetzlar, Germany) on cells stained for the microfilament F-actin and a nuclei counterstain with 4′,6-diamidino-2-phenylindole (DAPI), as described previously (Sieh et al., 2010). Live-dead staining was performed by incubating the cells at 37°C for 15 minutes with 3.35 μg/ml fluorescein diacetate and 25 mg/ml propidium iodide (both sourced from Life Technologies) diluted in serum-free/phenol red-free media prior to *z*-stack acquisition by CLSM.

### Animal experiments and BLI

All animal experiments were approved by the Queensland University of Technology Animal Ethics Committee (approval number 0900000915) in accordance with the *Australian Code of Practice for the Care and Use of Animals for Scientific Purposes*. Female NOD/SCID *Mus musculus* (mice) at 4 weeks old were purchased from the Animal Resources Centre (Canning Vale, Western Australia, Australia) and held at the Pharmacy Australia Centre of Excellence animal facility from the University of Queensland (Brisbane, Queensland, Australia). The animals were allowed to settle for 1 week prior to the subcutaneous implantation of one construct into each flank. Mineralized tissue formation occurred in 100% of hTEBCs and was monitored by X-ray analysis using an In-Vivo FX Image Station (Eastman Kodak Company, Rochester, NY) until sacrifice after 7 weeks (2 mice, *n*=4 hTEBCs) or 12 weeks (5 mice, *n*=10 hTEBCs).

For the experimental metastasis assay, 18 mice were implanted with hTEBCs and bone was allowed to form for 10 weeks *in vivo*. Mineralized tissue formation occurred in 100% of hTEBCs. Then 10^5^ BC cells suspended in 100 μl phosphate buffered saline (PBS) were injected into the left ventricle of the heart as described previously (Yoneda et al., 2001). One mouse died prematurely and was not included in subsequent analyses. Cancer cell dissemination was monitored weekly by *in vivo* BLI using a Xenogen IVIS Spectrum (PerkinElmer, Waltham, MA). Image acquisition was performed 15 minutes after intraperitoneal injection of 1.5 mg XenoLight D-Luciferin Potassium Salt (PerkinElmer). At the experimental end point, hTEBCs, mouse bones and organs were excised and *ex vivo* BLI was performed within 20–30 minutes after luciferin injection. Signals were quantified with the Living Image software (PerkinElmer) by drawing an automatic region of interest with a threshold set at 10% around each bioluminescent source to determine the amount of photons emitted for a given time. Only signals above 200 counts were considered positive. The take rates of metastasis to different tissues are indicated per mice, except where expressly stated for the hTEBCs (there were two constructs per mice). Explanted hTEBC and femur specimens were fixed in 4% paraformaldehyde overnight and then transferred to 70% ethanol until further analysis.

### *Ex vivo* μ-CT analysis

Scans of hTEBCs were taken using a μ-CT 40 scanner (Scanco Medical, Brüttisellen, Switzerland) at a voxel size of 20 μm, intensity of 145 μA and voltage of 55 kV. Samples were evaluated at a threshold of 150, a filter width of 0.8 and filter support of 1. X-ray attenuation was correlated to sample density using a standard curve generated by scanning hydroxyapatite phantoms with known mineral density. BV, BV/TV and BMD were quantified. Statistical analysis was performed using the SigmaPlot software (Systat Software, San Jose, CA). A Shapiro-Wilk normality test was performed. The statistical differences between groups were analyzed using a one-way analysis of variance for data that showed a normal distribution. Data that failed the normality test were analyzed using a Kruskal-Wallis one way analysis of variance on ranks. *P*<0.05 was considered significant.

### Histology and IHC

Fixed samples were decalcified for 5 weeks in 10% EDTA (pH 7.4) with weekly changes and subsequently embedded in paraffin. Sections near the central area of the implants were used for H&E staining and IHC analysis of the expression of human NuMA (dilution 1:100; S2825, Epitomics, Burlingame, CA), vWF (1:300; ab7356, Merck Millipore, Darmstadt, Germany), collagen type II (1:200; II-II6B3, DSHB, Iowa City, IA), human CD44 (1:50; H4C4, DSHB), human mitochondria (1:500; ab92824, Abcam, Cambridge, UK), human collagen type I (1:300; I-8H5, MP Biomedicals, Santa Ana, CA), human osteocalcin (1:200; ab13420, Abcam) and human CD146 (1:25; NCL-CD146, Leica Biosystems, Newcastle, UK). For IHC, de-waxed and rehydrated sections were incubated with 3% hydrogen peroxide (Sigma-Aldrich) for 30 minutes to block endogenous peroxidase activity. When targeting intracellular proteins, 6 minutes permeabilization with 0.1% Triton X-100 in PBS was performed. For antigen retrieval, the sections were either incubated with proteinase K (Dako, Glostrup, Denmark) for 30 minutes at room temperature or placed in trisodium citrate buffer (pH 6) for 4 minutes at 95°C, before blocking with 2% BSA (Sigma-Aldrich) for 60 minutes. The sections were incubated with the primary antibody solutions in blocking buffer overnight at 4°C, followed by 1 hour of incubation at room temperature with EnVision+ Dual Link System-HRP Rabbit/Mouse (Dako). Color development was performed with liquid diaminobenzidine chromogen (Dako) and sections were counterstained with Mayer’s hematoxylin (Sigma-Aldrich). Human tissue and mouse bone sections were used as positive and negative controls, respectively, for human-specific antibodies.

### TRAP staining

De-waxed and rehydrated sections were washed for 5 minutes in deionized water and incubated for 20 minutes at room temperature in 0.2 M acetate buffer consisting of 0.2 M sodium acetate (Sigma-Aldrich) and 50 mM sodium L-tartrate dehydrate (Sigma-Aldrich) in deionized water. Then, sections were transferred to acetate buffer containing 0.5 mg/ml naphthol AS-MX phosphate disodium salt (Sigma-Aldrich) and 1.1 mg/ml Fast Red TR salt 1,5-naphthalenedisulfonate salt (Sigma-Aldrich). Sections were incubated at 37°C for 2 hours until osteoclasts appeared red. Then, sections were rinsed in deionized water, counterstained with hematoxylin and mounted with an aqueous medium (Clear-Mount, ProSciTech, Thuringowa, Queensland, Australia).

## Supplementary Material

Supplementary Material
