# Supplementary material for: A tissue-engineered humanized xenograft model of human breast cancer metastasis to bone
Source: Dis Model Mech. 2014 Feb;7(2):299–309. doi: 10.1242/dmm.014076 (PMC3917251; doi:10.1242/dmm.014076)
Supplement: Supplementary Material [file supp_7_2_299__index.html]

A tissue-engineered humanized xenograft model of human breast cancer metastasis to bone — Supplementary Material 

# A tissue-engineered humanized xenograft model of human breast cancer metastasis to bone

## DMM014076 Supplementary Material

**Files in this Data Supplement:**

- **Supplementary Material PDF**
