# Supplementary material for: A tissue-engineered humanized xenograft model of human breast cancer metastasis to bone
Source: Dis Model Mech. 2014 Feb;7(2):299–309. doi: 10.1242/dmm.014076 (PMC3917251; doi:10.1242/dmm.014076)
Supplement: Supplementary Material [file supp_7.2.299_DMM014076.pdf]

Supplementary material

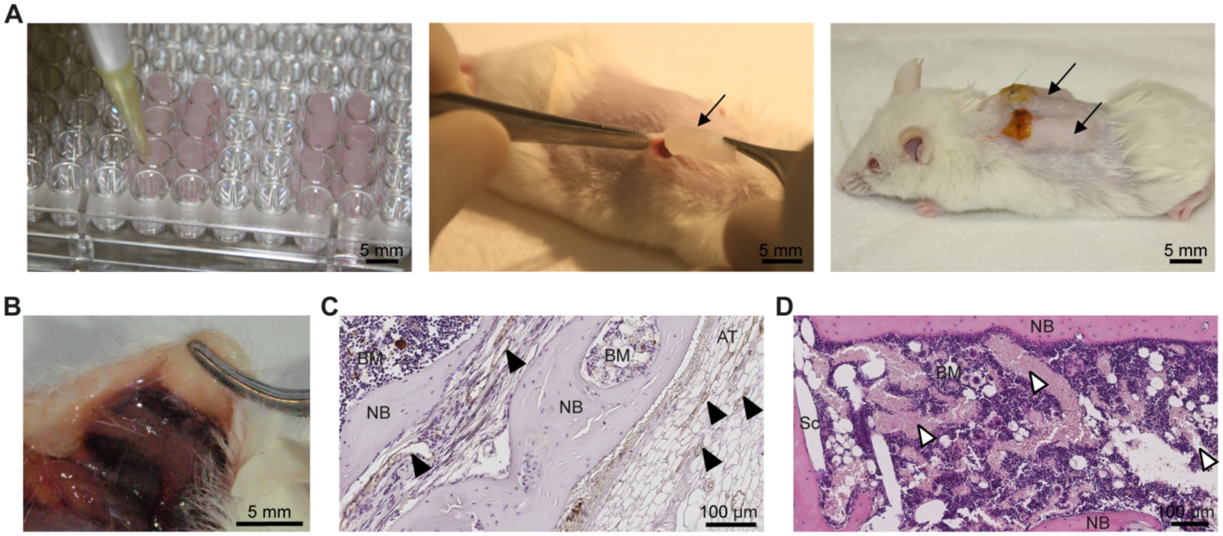

Figure S1: Implantation of hTEBCs leads to well vascularized constructs. A: Preparation and implantation of the hTEBCs (black arrow: hTEBC). B: Macroscopic image showing that the hTEBC is well integrated under the mouse skin and has a dark red color indicative of red proliferative bone marrow. C: Vascularization of the newly formed bone shown by the presence of vWF-positive blood vessels (brown stain, black arrow-heads). D: The bone marrow niche has an extensive vascular supply through a large sinusoid network (white arrow-heads). NB: new bone, BM: bone marrow, AT: adipose tissue, Sc: scaffold.

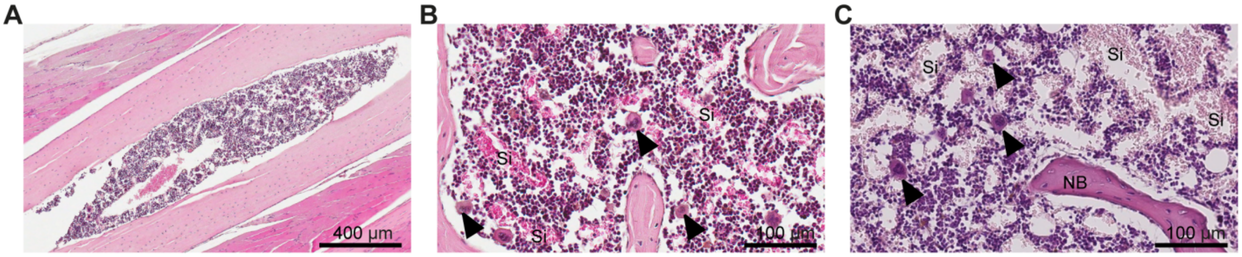

Figure S2: Similarity between the bone marrow found in the mouse tibia (A, B) and the engineered bone ossicle (C). In both types of samples the bone marrow shows areas of erythroid hematopoiesis, sinusoids filled with erythrocytes and megakaryocytes (black arrow-heads). NB: new bone, Si: sinusoids.

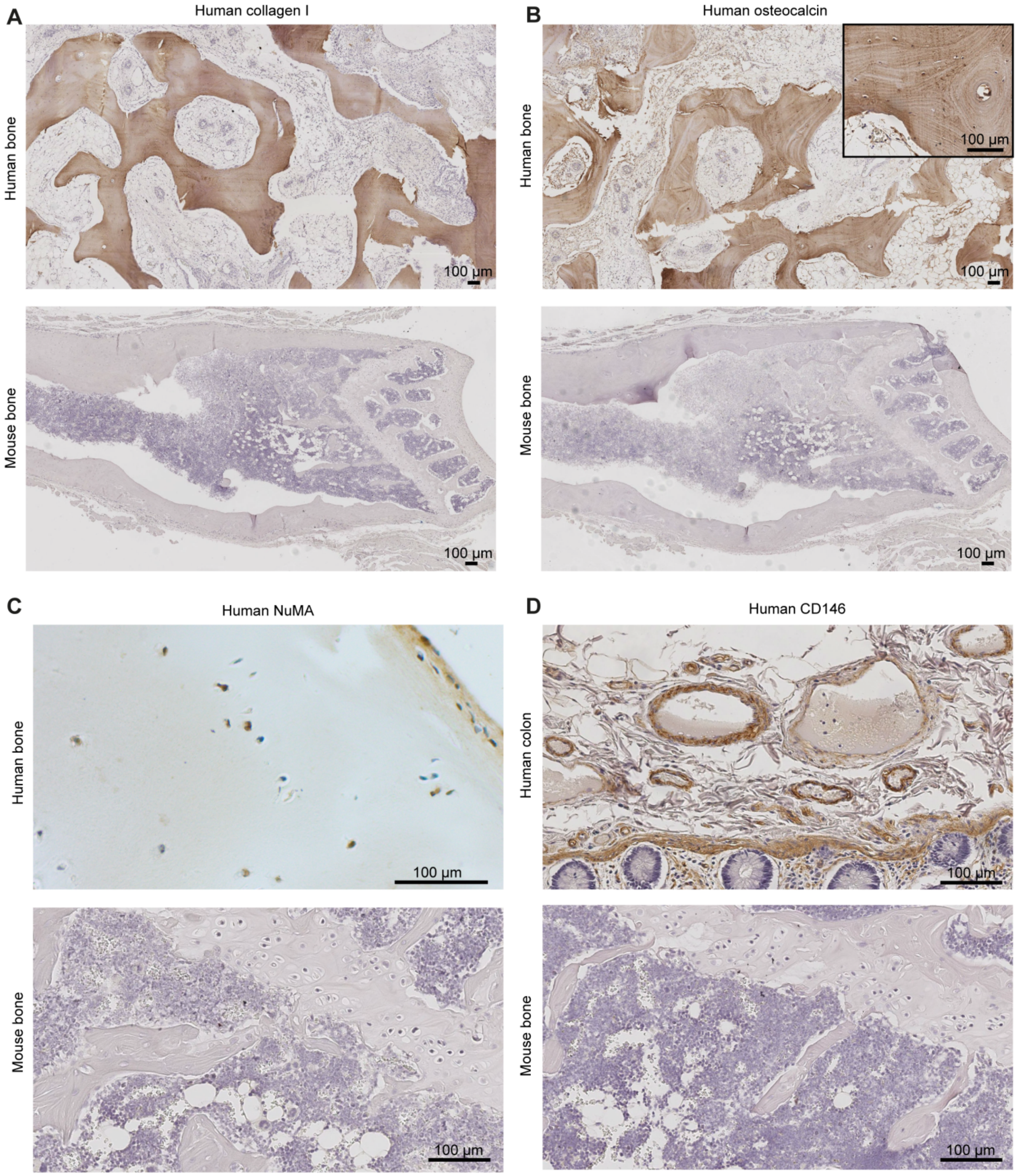

Figure S3: Human tissue and mouse bone were used respectively as positive and negative controls when testing the human-specific antibodies against collagen type I (A), osteocalcin (B), NuMA (C) and CD146 (D).
